# Measuring success in global health diplomacy: lessons from marketing food to children in India

**DOI:** 10.1186/s12992-016-0169-5

**Published:** 2016-06-16

**Authors:** Richard Smith, Rachel Irwin

**Affiliations:** London School of Hygiene & Tropical Medicine, Faculty of Public Health and Policy, 15-17 Tavistock Place, London, WC1H 9SH UK; Department of Public Health Sciences, Karolinska Institutet, Tomtebodavägen 18a, Solna, 171 77 Sweden

**Keywords:** Diplomacy, Marketing, Children, India

## Abstract

Global health diplomacy (GHD) focuses on international negotiation; principally between nation states, but increasingly non-state actors However, agreements made at the global level have to be enacted at the national, and in some cases the sub-national level. This presents two related problems: (1) how can success be measured in global health diplomacy and (2) at what point should success be evaluated? This commentary highlights these issues through examining the relationship between India and the WHO Set of Recommendations on the Marketing of Food and Non-alcoholic Beverages to Children, endorsed by Resolution WHA63.14 at the 63rd World Health Assembly in 2010.

## Commentary

### Introduction

Global health diplomacy (GHD) focuses on international negotiation, which includes a range of processes, from finalising agreements between multilateral or bilateral aid donors and recipient countries, to the processes of making binding and non-binding international agreements in health or related to health. In the latter case, which this commentary addresses, negotiations are principally between nation states. Although non-state actors may not be formally involved in negotiations, they are often and increasingly involved in consultations and other processes in the periphery. The ‘success’ of GHD is often seen as the production of an agreement, especially when formalised through international law, rather than improved health [1]. However, the ability of GHD to impact health relies on political purchase at the national, and often sub-national, level. This presents two challenges: (1) how and (2) when to measure the success of GHD.

This commentary examines the interaction between international agreements and the national-level, focussing on the WHO’s Set of Recommendations on the Marketing of Foods and Non-alcoholic Beverages to Children. It draws upon a wider ethnographic study [2], which included six months of participant observation at WHO headquarters (2010–2011) in the unit was responsible for drafting the Set of Recommendations. Over the course of this study (2010–2012), twenty-eight in-depth interviews on the process of the Set of Recommendations were conducted.. Interviewees included WHO staff, staff from other relevant UN bodies, member state representatives (including those on WHA and Executive Board delegations), representatives from NGOs and the private industry, including the advertising sector. For the case study on India, a further 10 interviews with key stakeholders were conducted in October 2010 in Delhi with representatives from the Ministry of Health, Consumers International (India), the Nutrition Foundation of India, Centre for Chronic Disease (India) and representatives from the pharmaceutical and food and beverage industry; the pharmaceutical industry was included as the portfolio of some companies includes non-alcoholic beverages. A further three respondents from these organizations, but not based in Delhi, were interviewed remotely. The interviews were semi-structured, with questions following organically according to responses being made and information being shared, but set to cover several broad topic areas: national context concerning obesity and marketing foodstuffs to children; relationship between Indian domestic politics and global agenda concerning health issues; relationships amongst key stakeholders; perspectives of and from key actors; perceptions of the impact of the Set of Recommendations on local governmental and non-governmental parties and the impact of other related WHO policies, such as the Global Strategy on Diet, Physical Activity and Health. This research was approved by the ethics review board of the London School of Hygiene and Tropical Medicine.

## Childhood obesity, food marketing and WHO recommendations

Childhood obesity is a global concern, associated with a variety of causal influences [3, 4], including the marketing of food and non-alcoholic beverages to children. In 2010 the 63rd World Health Assembly endorsed ‘The Set of Recommendations on the Marketing of Food and Non-alcoholic Beverages to Children’, urging member states to create policies to reduce the marketing of foods high in saturated fats, trans-fatty acids, free sugars, or salt [5]. Drafting the Set of Recommendations included consultation with WHO regions, dialogue with the private sector and non-governmental stakeholders, and the convening of an ad-hoc expert group. The Recommendations are non-binding, but represent official WHO policy and set out norms and standards. At one level, the Recommendations represent ‘successful’ GHD; at the time, representatives of all stakeholder groups were satisfied with the process and outcomes [2, 6–8] and the Recommendations, if implemented, would be expected to improve health. However a successful agreement at the international level does not translate into implementation at a national level; hence these desired health benefits may not materialise.

## The national not global context matters: India in focus

India comprises both very rich and very deprived populations, and obesity prevalence varies considerably by geography and socio-economic status. Childhood obesity is increasingly prevalent in children of higher socio-economic status, particularly the urban wealthy [9, 10]; between 2002 and 2007 there was an increase from 16 % to 24 % in urban children in Delhi, largely felt to be due to increased eating at fast food venues and television viewing [9, 10]. However, obesity often takes second place to the importance of under- and mal-nutrition and addressing childhood obesity is seen as somewhat of a ‘luxury’.

This creates potential dissonance between national and global priorities. This is exemplified in the idea that for most of India more calories rather than less is desirable, which is the antithesis of the Set of Recommendations. For instance, the Set of Recommendations refers to nutritional labels, with the idea that those high in calories and fat may be discouraged. However, as one respondent from the private sector indicated, in India ‘Diet Coke’ cans are printed with a warning label that they are not for children because the government does not feel that it would be appropriate for a child to consume a beverage with no calories. In another example, a respondent from a research institute stressed that there is a perception, particularly in Northern India, that it is healthy to be obese because ‘thin children die.’

Thus the Set of Recommendations were felt not to address the specific social and cultural factors relating to India, which is a significant barrier to successful implementation, as well as their engagement with the drafting process. When asked about risk factors for childhood obesity, respondents listed a number of issues they perceived to cause obesity: as more women enter the workforce, there is the perception that women are choosing convenient (less healthy) options for family meals; western-made goods, which are highly processed and less healthy, are seen as superior, and often safer, with one respondent stating that ‘it is in the Indian psyche…will do anything to substitute the natural’; increasing emphasis on academic performance at school was also felt to perhaps limit access by children to sport and physical activity as households become wealthier; the increased popularity of processed food, use of formula in infancy, lack of choice in school canteens, unhealthy packed lunches and consumption of fried street food, which is high in transfatty acids, are also all major changes in Indian lifestyles. (See also [9]).

It was also felt that there was inadequate consideration in the Set of Recommendations of obesity in India as being concentrated in the wealthy. One respondent from a research institute remarked that the scientific community – both inside India and externally – was ‘in a rut’ and ‘trapped in the thinking that India equalled poor and malnutrition.’ Similarly, a respondent from the private sector noted that there was little data on the issue and what existed was unmatched, unpublished and ‘scattered.’

Indeed, negotiations in global health diplomacy are highly reliant on clinical or epidemiological evidence and the quality or type of evidence can either constrict or expand policy options. The experience of low-and-middle income countries was missing from much of the process of the Set of Recommendations. For example, the WHO ad hoc expert group considered two systematic reviews on The Extent, nature, and effects of food promotion to children [11, 12]; the more recent document was an update of the first. In these, one hundred fifteen studies met the inclusion criteria. Of these, only ten studies included a component on countries outside of Europe, the US, Canada or Antipodes, four of which examined India.

The authors of the studies were well aware of this limitation and tried to mitigate it. For example, in the first review, researchers conducted supplemental desk research using business and marketing press, journals and commentaries from non-governmental organisations to map the marketing environment in low-and-middle income countries In the latter review, there is an entire section devoted to ‘food promotion and marketing in developing and middle income countries’ which pulls out more detailed data from the 10 applicable studies. Additionally, there was geographic diversity in the ad hoc expert group.

This lack of input from low and middle-income countries was also found in the countries and organisations represented in the stakeholder dialogue, few of whom had experience in low or middle-income countries. There is a sense that global representation is a goal at the WHO, but there are few health issues that are evenly distributed across the globe. Cairns et al. found that food companies in middle-income countries used similar marketing techniques as in high-income countries, but had very little data on low-income settings. This meant that in the final version of the Set of Recommendations there was greater focus on television and Internet advertising and less on advertising methods in low incomes countries, such as billboard, print and point-of-sale.

There was concern that using a ‘Western’ view of the problem was inadequate for interventions, which related to wider critiques about the drafting process focussing on the experience of high-income countries. For instance, because of the disparity in wealth, firms target different classes in different ways, and several respondents gave the example of firms selling “5 rupee” products – products that are packaged in smaller portions and sold at more affordable prices, which parallels the experience in tobacco sales. Thus any policies concerning food and food sales would need to consider the many ways in which food is sold. Further, India reflects starkly the reality that any country is several markets rather than one, with a wide variety of types of marketing – including those that may reach children. Thus, limiting marketing via television, radio and mobile phones may address the types of sponsorship found in wealthy areas, but not those more common in poorer areas, including billboards or sponsorship of schools or sporting events.

Overall, respondents felt that the disparity in importance of the health burden to most of the population, and in the legislative capacity, and indeed priority, to tackle what is seen as a minor issue, made the Set of Recommendations feel of less, if any, relevance to government. Non-communicable disease in general has only recently reached the political agenda, and the Ministry of Health is just now rolling-out its first non-communicable disease programme (based on the WHO’s Global Strategy for Diet, Physical Activity and Health). Although at the time of the study, respondent reported that the Ministry planned to hire a number of people at headquarters and throughout the country who would be associated with this programme, until then non-communicable disease is dealt with on a national level with by a team of 4–5 people in a country with over one billion inhabitant. According to a Ministry representative, there is a wide disjuncture between the global context within which the Recommendations were formulated, and the specific national context; an expression of a more general limitation of global health diplomacy that no one policy will be fully applicable to all country contexts

## Discussion

Is success in GHD measured at the time of endorsing Recommendations, or in their implementation? All ‘global’ health diplomacy has to be played out through national politics. Thus, a successful conclusion to international negotiations is part of a process, not the end, and a consideration of barriers and issues concerning national action is an important part of conducting and evaluating GHD.

Within global health, there is examination of diplomacy involved in setting the stage for the development of agreements, and around the negotiation of agreements, but less reflecting the interface of this global level diplomacy and the national context within which these agreements are implemented. The Indian case presented here offers two lessons.

First, despite the discussion of ‘new diplomacy’ going beyond nation states, it is nation states which are ultimately responsible and able to implement recommendations. Indeed, according to the Set of Recommendations “Governments should be the key stakeholders in the development of policy and provide leadership, through a multi-stakeholder platform, for implementation, monitoring and evaluation. Thus, although governments may choose to work with other sectors and stakeholders, they are “in the best position to set direction and overall strategy to achieve population-wide public health goals” [5] Ensuring that agreements appeal to national agendas or can provide tools to enable national agendas to be realigned by others is therefore critical. This also includes recognising differences in national-level public health governance: what may be the responsibility of a ministry of health in one country may fall under a different ministry in another.

Second, global norms and standards will never fully match the contexts within individual countries. Indeed, this is deliberately the case, as global agreements are usually written quite broadly, so they may be adopted. They are also often aspirational, representing a norm towards which countries can work, although the extent to which individual governments accept global norms depends on the national context. The potential for an agreement, particularly a non-binding one, to be applicable to 194 Member States, is likely unrealistic.

That India did not take immediate steps to implement the Recommendations, nor to address marketing of food to children more widely, does not represent a failure of GHD. Rather, it reflects the reality of making global policy applicable to the individual contexts of 194 WHO Member States, where the adoption of norms takes time and that the implementation of global health diplomacy outcomes must be conducted within national contexts and limitations. For example, although the WHO Global Strategy for Diet, Physical Activity Health was adopted in 2004, it was not until six years later that India had finalised its own NCD strategy, which was a direct response to the WHO Strategy. Moreover, despite the reliance on governments as key players, in many cases in which a government is not seen to be prioritising an issue, we see action from non-state actors in both implementation and monitoring of agreements.

## Conclusions

Overall, the lesson from this case study is that those involved in research and action concerning GHD need to develop means to evaluate both the appropriate range of ‘outcomes’ of the diplomatic activity, and also to determine over what time period this evaluation is to occur. Methodologically it is also necessarily, but difficult, to focus on a non-case study. For example, studying Norway’s implementation of the Set of Recommendations would have been much easier than India, as Norway was highly involved in the process leading up to their endorsement. However, in studying the implementation of GHD is it just as important, although more challenging, to examine the cases in which implementation appears lacking*.* Without this focus on the examples that demonstrate a lack of implementation, there is no means to understand whether a specific diplomatic activity has been worth the effort, and what factors have contributed to this, to inform further diplomatic developments for health. That this is important is illustrated by the conundrum posed by India and the WHO Recommendations: has this example of GHD ‘failed’ because India did not appear to act upon it? At present we are limited to reporting a snapshot in the process, but evaluation judgements are hard to make and will continue to be so for other cases unless progress is made in this methodological area.
